# Establishment and Clinical Significance of the Patient-Derived Xenograft Model of Colorectal Cancer

**DOI:** 10.7759/cureus.71116

**Published:** 2024-10-08

**Authors:** Li Zhang, Yuhong Li, Liuxu Yao, Rui He, Jianqiang Wu

**Affiliations:** 1 Anesthesiology, Hangzhou Linping Qiaosi Community Health Service Center, Hangzhou, CHN; 2 Anesthesiology, Zhejiang Shuren University, Hangzhou, CHN; 3 Anesthesiology, Zhejiang People’s Hospital, Hangzhou, CHN; 4 Anesthesiology, Shaoxing People's Hospital, Shaoxing, CHN

**Keywords:** colorectal cancer, drug intervention, influencing factors, patient-derived xenograft (pdx), propofol

## Abstract

Objectives: Patient-derived xenograft (PDX) models are widely acknowledged for their ability to reflect the heterogeneity of human cancers and can be used to improve preclinical models. In this study, we evaluated the factors affecting the tumor formation rate of the PDX colorectal cancer (CRC) model and conducted preliminary drug sensitivity tests.

Methods: CRC patients who underwent elective surgery at Shaoxing People's Hospital from November 2019 to October 2020 were included. The tumor tissue obtained from surgery was transplanted to the back of NSG mice, and the PDX model was established and subcultured to the F3 generation. Factors that affected tumorigenicity were analyzed and compared histologically. Drug interventions included 5-fluorouracil, oxaliplatin, and propofol.

Results: Sixty CRC patients were included in this study, and tumorigenesis was observed in CRC tissue derived from 37 cases (62%). The primary tumor malignancy degree (tumor stage and degree of cell differentiation), preoperative carcinoembryonic antigen level, and tumor location in CRC patients could affect the tumorigenicity of the PDX model. Histopathological analysis of CRC-PDX transplanted tumor tissue was highly consistent with the patient's tumor tissue. All four chemotherapy regimens could inhibit tumor growth and cause tumor tissue damage. Propofol could inhibit diarrhea in mice and protect intestinal mucosa.

Conclusions: The CRC-PDX model established in this study can maintain the biological characteristics of primary tumors and can be used as a reference model for the individualized treatment of CRC patients. The degree of malignancy of the primary tumor is the primary factor affecting the tumorigenesis rate of the PDX model.

## Introduction

Colorectal cancer (CRC) is one of the most common malignant tumors. In recent years, the incidence and mortality of CRC have increased following changes in our dietary and lifestyle habits [1]. For most patients with advanced CRC, surgery alone is not curative. Chemotherapy and targeted drug therapy are important means to improve treatment efficacy. Indeed, it is well-established that understanding the heterogeneity of CRC is essential to find an optimal therapeutic approach to reduce morbidity and mortality [2]. Genome-wide analysis has shown that each CRC has a median of 76 non-silent gene mutations [3], and each CRC case is unique. The high failure rate of clinical trials of chemotherapy drugs can be attributed to some extent to the limitations of preclinical models [4] and the poor ability to predict the prognosis of patients treated with candidate drugs. The traditional cell line-derived xenograft (CDX) model involves the culture of human tumor cells in vitro, subcultured to establish a stable cell line, and then injected into immune-deficient mice. However, during tumor cell culturing, the biological characteristics of the primary tumor and the tumor heterogeneity are lost [5]. The patient-derived xenograft model (PDX) developed in recent years involves transplanting fresh tumor tissue from patients to immunodeficient mice and the subsequent growth of tumor tissue in the environment provided by mice [6]. The PDX model is the closest tumor model to clinical research so far, which has important transformation significance for tumor preclinical evaluation, treatment, and prognosis, and is expected to bring breakthroughs for individualized treatment of tumor patients. The PDX model provides a valuable tool for understanding the potential mechanisms of intercellular communication, drug response, and tumorigenesis and for drug discovery and individualized treatment. Among various animal models of CRC, the PDX model retains the characteristics of tumor tissue of patients, including histopathological structure, genomic characteristics, clonal heterogeneity in the tumor, chromosome instability, and drug reactivity. Indeed, mutations of key driving genes remain consistent following several cell passages [7,8]. CRC-PDX has many limitations for large-scale clinical use, among which PDX tumorigenesis rate and influencing factors are important limiting parameters.

Anesthetics may affect the occurrence and development of cancer through the activation of the immune system, adrenergic inflammatory pathway, and tumor suppression signal pathways [9]. Propofol is a commonly used anesthetic. Many studies have shown that propofol can inhibit tumor growth and improve the prognosis of tumor patients [10,11].

NF- κB, HIF-1α, and Ki67 are key genes related to the occurrence and development of CRC. To explore the morphological and genetic stability of the PDX model, this study established a CRC-PDX model, analyzed each generation of tumor tissue, and identified the stability of this model, laying a foundation for future in vivo drug research. This study was divided into two parts. First, CRC tumor tissue was inoculated in NSG mice to construct the CRC-PDX model; the tumor formation rate and the factors influencing tumorigenesis were analyzed. In the second part, PDX model mice were subcultured to F3 generation to explore the stability of tumor morphology and biological activity after three generations and preliminary drug experiments were carried out to explore the values of the PDX model for drug screening of clinical tumor patients.

## Materials and methods

Experimental animal

Immune-deficient NSG male SPF mice, 7-8 w, weight 18-24 g, were purchased from Jiangsu Jicui Yaokang Biotechnology Co., Ltd. (code of production certificate of experimental animals: SYXK (Su) 2018-0008). The experiments were conducted at the Animal Laboratory of Shaoxing People's Hospital (license number is SYXK (Zhe) 2017-0007). Animals were raised in an IVC environment for one week under controlled conditions (room temperature of 26 ℃±1 ℃, 50%-60% humidity, 12hrs: 12hrs light/dark cycle, ventilation rate of 8-15 times/h) and the growth of mice was observed every day. The present experiment was performed according to an approved animal protocol approved by the Animal Ethics Committee of Shaoxing People’s Hospital (No. 2020-114). The study is reported in accordance with the ARRIVE guidelines for reporting experiments involving animals [12].

Clinical specimen

All methods were carried out in accordance with the Declaration of Helsinki. The research protocol was reviewed and approved by the Ethics Committee of Shaoxing People's Hospital (Ethics No. 2017-145). The patient and his family members were informed that the patient’s privacy would be protected when participating in the study, and the specimens removed from surgery would be kept in the hospital and possibly used for scientific research. This protocol was registered in the Chinese clinical trial registry (registration number, ChiCTR1900026975). 60 patients with colorectal cancer who underwent elective colorectal cancer surgery in the Anorectal Department of Shaoxing People's Hospital from November 1, 2019 to October 1, 2020, were recruited in this study. The inclusion criteria included: Pathological diagnosis of colorectal cancer, tumor size of more than 4 cm, primary cancer or metastatic cancer. Patients with HIV patients, hepatitis B patients, or hepatitis C were excluded from the study. During the surgery, fresh tissue samples were harvested under sterile conditions. Three pairs of tumor tissues in situ and three pairs of metastatic tumor tissues were taken as parents (P) with a sample volume of at least 6 mm×6mm×6 mm (>200 mm^3^). One pair was put into a tissue preservation solution for constructing the PDX model, and the other two pairs were placed in 10% formalin fixative for morphological and immunohistochemical studies.

Main reagents

Sufentanil citrate injection (Yichang Renfu Pharmaceutical Co., Ltd.); Sevoflurane, oxaliplatin (Jiangsu Hengrui Pharmaceutical Co., Ltd.); propofol (AstraZeneca UK); 5-Fluorouracil (Tianjin Jinyao Amino Acid Co., Ltd.); magnetically activated cell sorting (MACS) tissue sample preservation solution (Germany Meitianni Biotechnology Co., Ltd.); double antibody (penicillin, streptomycin), 4% paraformaldehyde, diluent of first antibody and HE staining kit (Shanghai Biyuntian Co., Ltd.); Ki67 antibody, HIF-1 α antibodies, and NF- κB antibody (American Affinity Co., Ltd.) were used.

Establishment and passage of the PDX model of CRC

The tumor tissues of 65 CRC patients were harvested and termed parental generation (P). They were inoculated subcutaneously in the right lumbar back of 65 NSG mice to construct a CRC-PDX mouse model (P0 generation). When the tumor volume reached 1 cm^3^, rapid decapitation was used to sacrifice the mice, then the tumor tissues were harvested. Some tissues were used for passage, and some were placed in 4% paraformaldehyde. The P0 generation tumor tissues were inoculated into 3-5 NSG mice to construct the F1 generation CRC-PDX model. When the tumor volume reached 1 cm^3^, the mice were killed by rapid decapitation and the tumor tissues were retained for passage or further research. The volume was calculated as follows: tumor volume=(maximum tumor diameter × minimum tumor diameter^2)/2. By analogy, it was passed down to the F3 generation. The definition of tumor formation time (d): the time from the tumor tissue inoculation of P0 generation NSG mice to the tumor volume of 1 cm^3^. The observation time was set to 60 days in this study [13].

Immunohistochemical staining

5 μm of paraffin-embedded tissue block was baked at 60℃ overnight. After dewaxing with xylene, dehydration with tap water, and dehydration over a gradient of alcohols, tissues were incubated with 3% H_2_O_2_ for 10 min to eliminate endogenous peroxidase activity, antigen was repaired by microwave irradiation for 10 min and incubated in 1:100 mouse anti-HIF-1α, NF-κB, Ki67 polyclonal antibody (Invitrogen), overnight at 4 ℃. After washing with 0.1 mo1/L phosphate buffer solution (PBS) and they were subjected to immunohistochemical staining, 3',3'- diaminobenzidine (DAB) coloration, hematoxylin counterstaining, and blocking. Under the microscope, the nucleus was stained blue, and the gene expression positive area was brown or brown-yellow. The expression of HIF-1α, NF-κB, ki67 in the F3 generation of CRC-PDX transplanted tumor and the primary (parent) tumor tissue was compared and analyzed to determine whether CRC-PDX could replicate the biological characteristics of the parent tumor. The immunohistochemical score (IRS) was used for semi-quantitative determination (formula: IRS=SIPP) based on the staining intensity (SI) (0, no color development; 1 point, light yellow; 2 points, brown yellow; 3 points, dark brown) and percentage of stained cells (PP) (0, no color; 1 point, stained cells<10%; 2 points, stained cells 11%-50%; Grade 3, 51%-80% of stained cells; Grade 4, staining cells>81%).

Hematoxylin-eosin (HE) staining

HE staining was used to detect the morphological changes of intestinal mucosa in rats: the morphological changes of intestinal mucosa were observed under light microscopy with conventional HE staining, and the morphological characteristics of P versus F3 tumors were compared.

Study on drug sensitivity of the CRC-PDX model

Thirty F3 generation PDX model mice (from the same patient) were divided into five groups (n=6) according to the medication scheme: Control (Ctrl) group: 0.9% sodium chloride 100 µL; Propofol (Prop) group: Propofol 40 mg/kg; 5-fluorouracil (5-Fu) group: 5-fluorouracil 23 mg/kg; 5-fluorouracil combined with oxaliplatin (5-Fu+OX) group: 5-Fu 23 mg/kg combined with oxaliplatin 5 mg/kg; 5-Fu+Prop group: 5-Fu 23 mg/kg combined with Prop 40 mg/kg. All drugs were diluted to 100 µL with 0.9% sodium chloride and injected intraperitoneally once every other day, the tumor volume of mice was measured every three days, and activity, mental status, emaciation, and diarrhea of mice were observed. After two weeks of administration, the mice were killed by rapid decapitation, and the tumor tissues were harvested to measure the volume and mass. The tumor tissue was fixed in 4% paraformaldehyde for morphological or immunohistochemical detection.

Statistical analysis

 IBM SPSS Statistics for Windows, Version 18 (Released 2009; IBM Corp., Armonk, New York, United States). and GraphPad Prism 7 (GraphPad Software, California, USA) were used for statistical analysis. Dose data conforming to the normal distribution were expressed as mean (SD), and a student’s t-test was used to compare data between groups. The categorical variables were expressed by rate, and a χ2 Test or Fisher exact test was used to compare the difference of clinical and pathological factors between the tumor-formation group and non-tumor formation group, and multivariate logistic regression analysis was conducted for variables with P<0.05 to analyze the independent risk factors affecting tumor formation.

## Results

CRC-PDX model construction and generation

**Figure 1:**
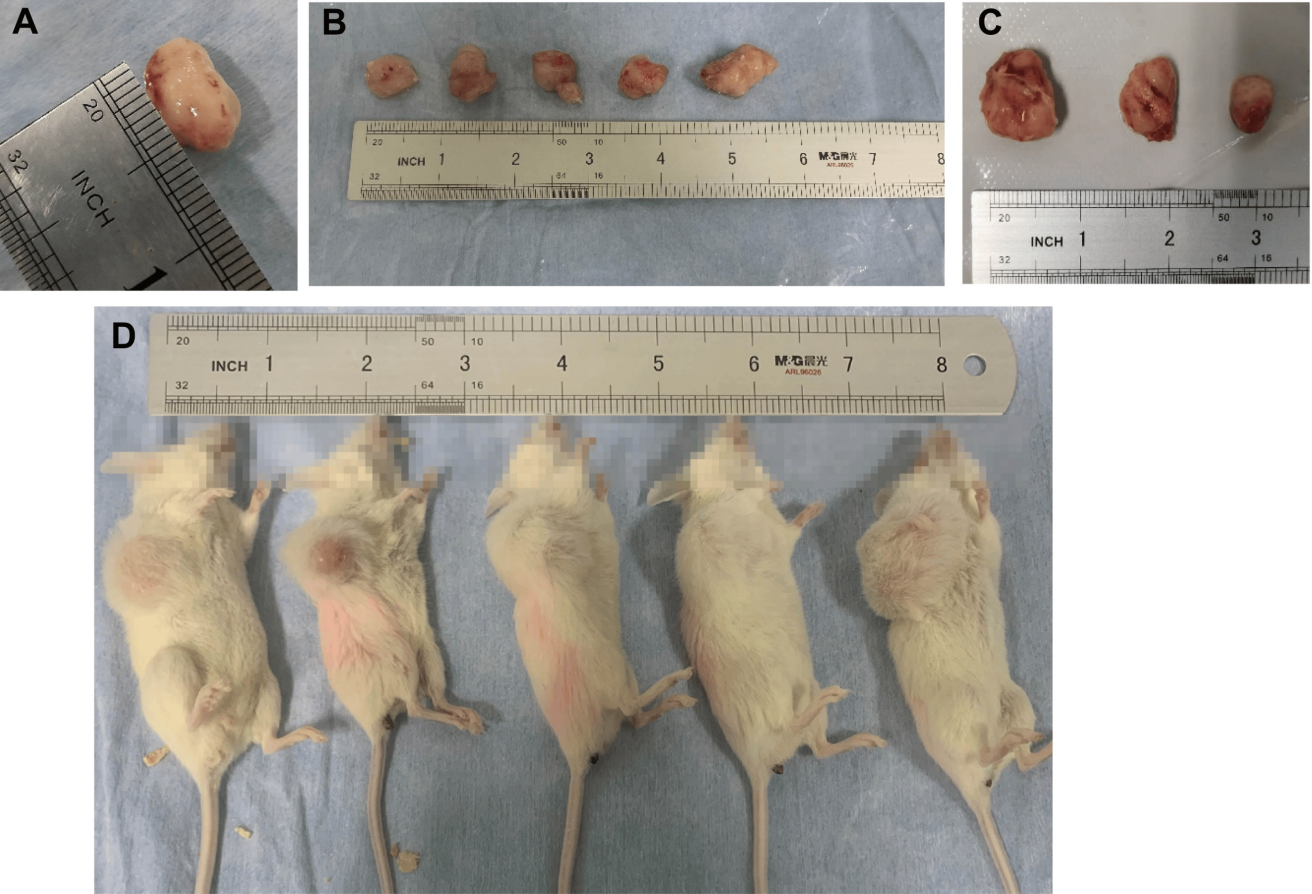
Tumorigenesis in CRC-PDX model mice CRC: colorectal tumor; PDX: xenotransplantation of tumor tissue from patients; A-C: represent the tumor tissue of P0, F1 and F2 PDX model mice respectively; D: F2 generation CRC-PDX model mice.

In this study, 65 patients with CRC were enrolled. Tumor tissues from patients were collected and inoculated into NSG mice to construct 65 P0 generation PDX model mice. Among them, five animals died, with a mortality of about 8%. Finally, 60 PDX model mice were included in the data analysis. The mean tumor formation time was 34(11) days, ranging from 10 to 58 days. Tumorigenesis was observed in 37 (62%) of the P0 generation PDX model mice (Figure 1).

Biological characteristics of tumor tissue and original tumor tissue of the CRC-PDX model

**Figure 2:**
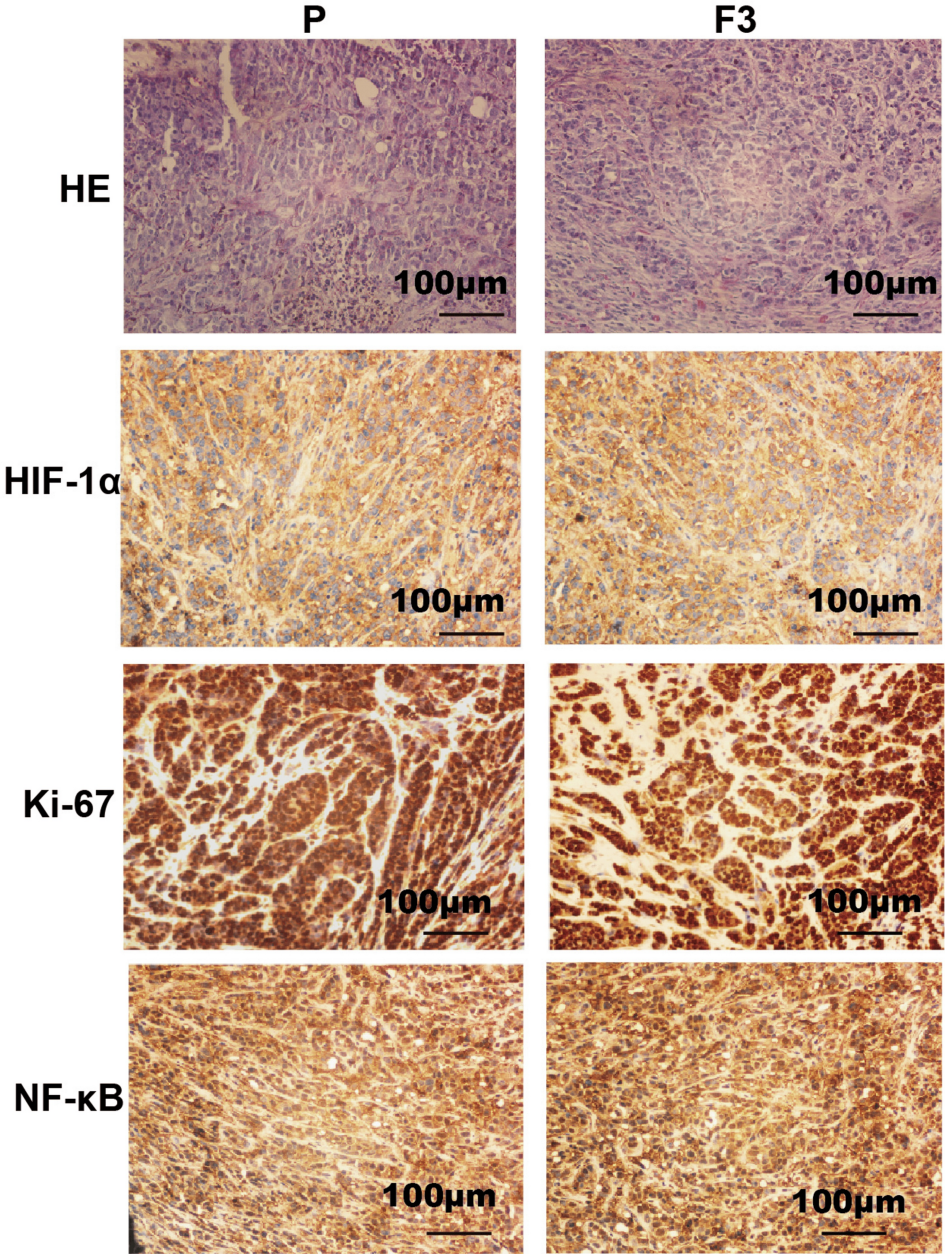
Comparison of HE staining morphology and expression of characteristic proteins between PDX model tumor tissue (F3) and patient's original tumor tissue (P)(×200) CRC: colorectal tumor; PDX: xenotransplantation of tumor tissue from patients; HE: hematoxylin and eosin staining

HE staining was performed on P and F3 generation tumor tissues. The results showed that the tissues of PDX model mice (F3) were highly consistent with the primary tumor tissues (P) in terms of cell tissue structure, and there were no significant changes in cell density, karyotype, and mesenchymal cell morphology, which preserved the histological characteristics of the primary tumor (Figure 2). Immunohistochemical staining was used to assess Ki67, NF-κB, and HIF-1α expression P versus F3 generation tumor tissues. A high expression (>80%) was observed, suggesting that during the passage of the PDX model, the histomorphology and tumor molecular phenotype of P versus F3 generation tumors remained genetically stable and the biological characteristics of primary tumors were well preserved (Figure 2).

Analysis of factors influencing the tumor formation rate

Our PDX models were derived from 60 CRC patients, mostly males (n=40, 66.7%), with a mean age of 63 (10) yrs, mean weight of 62 (10) kg, and mean height of 164 (8) cm. According to the tumor stage, the 60 CRC patients had stage I (n=10, 16.7%), stage II (n=15, 25.0%), stage III (n=16, 26.7%) and stage IV (n=19, 31.7%). Stratification based on cell differentiation showed that the PDX models were derived from highly differentiated (n=23,38.3%), moderately differentiated (n=22,36.7%), and poorly differentiated (n=15,25.0%) CRC. The most common metastatic sites in the CRC patients included liver (n=22,36.7%), lymph node (n=25,41.7%), and hematogenous (n=23,38.3%) metastasis. The tumorigenesis rate of models based on tumor tissues from patients with late-stage CRC was significantly higher (P=0.002), with 18 cases (94.7%) associated with stage IV tumors compared to 4 (40.0%), 7 (46.7%), and 8 (50.0%) in stage I, II, and III tumors, respectively. The degree of cell differentiation also significantly affected the tumorigenesis rate (P<0.001). Higher tumorigenesis rates were associated with moderately to poorly differentiated tissues. The tumorigenesis rates of highly, moderately, and poorly differentiated tissues were 21.5% (5/23), 77.3% (17/22), and 100% (15/5), respectively. Patients could also be stratified according to the preoperative CEA levels into <10 ng/mL (n=35, 58%) and ≥10 ng/mL (n=25, 42%) groups. A significantly higher tumorigenesis rate was observed in the <10 ng/mL groups (14/35, 40%) than in the ≥10 ng/mL group (n=23/25, 92.0%) (P<0.001). Besides, a significant difference was observed based on tumor site, with 19 (19/24, 79.2%), 9 (9/12, 75.0%), and 9 (9/24, 37.5%) cases of tumorigenesis in left colon cancer, right colon cancer, and rectal cancer, respectively (P=0.008). Factors including warm ischemia time, tumor metastasis, age, body weight and height did not affect the tumor formation rate (all P>0.05). Multivariate analysis was conducted to identify influential factors from significant parameters from univariate analysis. In view of the large error, the multivariate analysis was abandoned (Table 1).

**Table 1:** Patient characteristics and tumorigenicity of primary tumor in PDX models Data are expressed as the mean (SD), number of patients or median (Q1, Q3); PDX, patient-derived xenograft; CEA, carcinoembryonic antigen; WD, well differentiated; MD, moderately differentiated; PD, poorly differentiated; WIT, warm ischemia time.

	Patients	Tumorigenicity		
		Yes	NO	P-value
	(n = 60)	(n=37)	(n=23)	
Age, n (%)				0.402
<60 years	20 (33.3%)	14 (70.0%)	6 (30.0%)	
≥60 years	40 (66.7%)	22 (55.0%)	18 (45.0%)	
Gender, n (%)				0.511
Male	40 (66.7%)	23 (57.5%)	17 (42.5%)	
Female	20 (33.3%)	14 (70.0%)	6 (30.0%)	
Body weight (kg)	62 (10)	63.3 ( 8.1)	59.9 (8.0)	0.114
Height (cm)	164 (8)	164.0 (8.5)	163.0 (8.2)	0.399
Preoperative CEA level				<0.001
<10 ng/mL	35 (58.3%)	14 (40.0%)	21 (60.0%)	
≥10 ng/mL	25 (41.7%)	23 (92.0%)	2 (8.0%)	
Primary tumor location, n (%)				0.008
Right colon	12 (20.0%)	9 (75.0%)	3 (25.0%)	
Left colon	24 (40.0%)	19 (79.2%)	5 (20.8%)	
Rectum	24 (40.0%)	9 (37.5%)	15 (62.5%)	
Tumor stage, n (%)				0.002
I	10 (16.7%)	4 (40.0%)	6 (60.0%)	
II	15 (25.0%)	7 (46.7%)	8 (53.3%)	
III	16 (26.7%)	8 (50.0%)	8 (50.0%)	
IV	19 (31.7%)	18 (94.7%)	1 (5.3%)	
Cell type, n (%)				<0.001
WD	23 (38.3%)	5 (21.7%)	18 (78.3%)	
MD	22 (36.7%)	17 (77.3%)	5 (22.7%)	
PD	15 (25.0%)	15 (100.0%)	0 (0.0%)	
WIT, n (%)				0.787
<10 min	37 (61.2%)	14 (37.8%)	23 (62.2%)	
≥10 min	23 (38.3%)	10 (43.5%)	13 (56.5%)	
Lymphatic invasion, n (%)				1.000
Negative	35 (58.3%)	22 (59.5%)	13 (56.5%)	
Positive	25 (41.7%)	15 (40.5%)	10 (43.5%)	
Liver invasion, n (%)				0.607
Negative	38 (58.3%)	22 (57.8%)	16 (42.1%)	
Positive	22 (36.7%)	15 (68.1%)	7 (31.8%)	
Vascular invasion, n (%)				0.472
Negative	37 (61.7%)	21 (56.8%)	16 (43.2%)	
Positive	23 (38.3%)	16 (69.6%)	7 (30.4%)	
Xenograft number				0.073
1	15 (25.0%)	9 (60.0%)	6 (40.0%)	
2	15 (25.0%)	9 (60.0%)	6 (40.0%)	
3	15 (25.5%)	13 (86.7%)	2 (13.3%)	
4	15 (25.5%)	6 (40.0%)	9 (60.0%)	

Chemotherapy drug in sensitivity testing in CRC-PDX model mice

**Figure 3:**
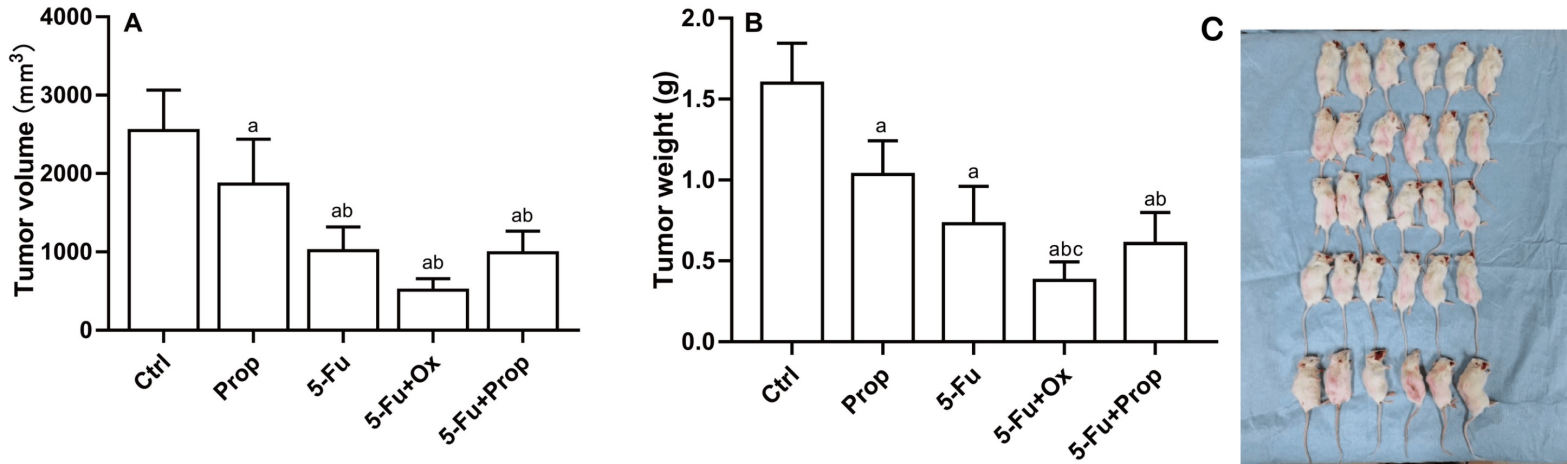
Changes in tumor volume and mass in F3 generation mice of the CRC-PDX model after different drug interventions Data are expressed as the mean (SD); CRC: colorectal tumor; PDX: xenotransplantation of tumor tissue from patients; A: tumor volume; B: tumor weight; C: PDX mice killed after drug intervention using rapid decapitation. ^a^P<0.05 compared with Ctrl; ^b^P<0.05 compared with Prop; ^c^P<0.05 compared with 5-Fu.

**Figure 4:**
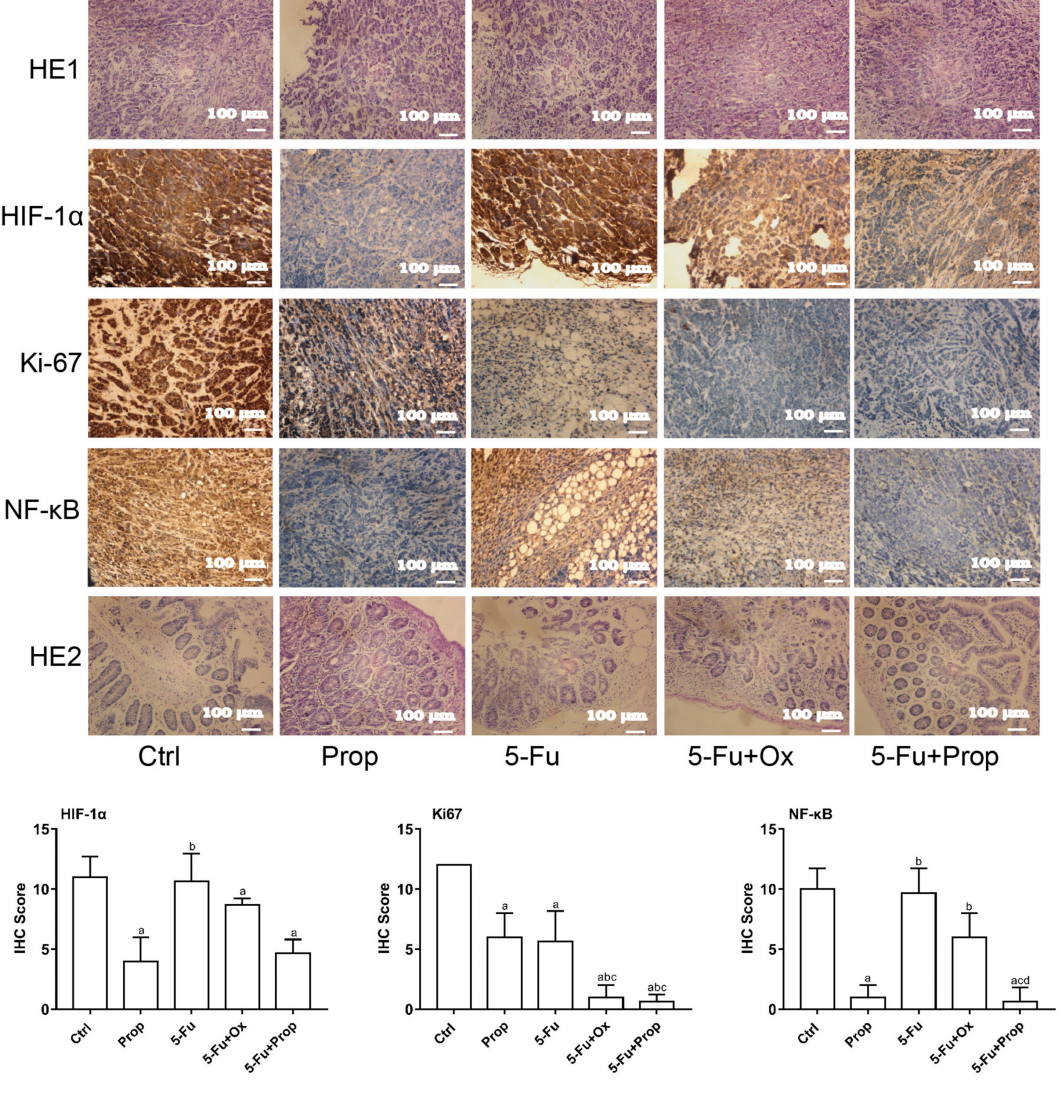
Pathological morphology of tumor tissue (HE1) and intestinal mucosa (HE2) and expression of tumor tissue characteristic proteins in F3 generation mice of the CRC-PDX model after different drug intervention (×200) Data are expressed as the mean (SD); CRC: colorectal tumor; PDX: xenotransplantation of tumor tissue from patients; HE1: HE: Hematoxylin and eosin staining of tumor tissue; HE1: Hematoxylin and eosin staining of the intestinal mucosa. ^a^P<0.05 compared with Ctrl; ^b^P<0.05 compared with Prop; ^c^P<0.05 compared with 5-Fu; ^d^P<0.05 compared with 5-Fu+Ox.

After two weeks of chemotherapy, the animals were killed by rapid decapitation (Figure 3C). Diarrhea occurred in mice in the 5-Fu group and 5-Fu+Ox group during chemotherapy; while no diarrhea occurred in the Ctrl group, Prop group, and 5-Fu+Prop group. The tumor volume and mass after four chemotherapy regimens were smaller than the Ctrl group (P<0.05). The 5-Fu+Prop group experienced a stronger anti-tumor effect than the Prop group (P<0.05). The 5-Fu group experienced a stronger anti-tumor effect than the Prop group (P<0.05). Importantly, the 5-Fu+Ox group sustained a stronger anti-tumor effect than the 5-Fu group (P<0.05) (Figures 3A, 3B). After treatment with four different chemotherapy schemes, tumor tissue exhibited varying degrees of damage during histopathological analysis (Figure 4). Immunohistochemical staining analysis of the expression of tumor-specific proteins such as HIF-1α, NF-κB, and Ki67 showed that Prop could significantly inhibit the expression of three proteins (P<0.05). A significantly stronger inhibitory effect was observed in the 5-Fu+Prop combination group on Ki67 than in the Prop monotherapy group (P<0.05). Compared with the Ctrl group, the 5-Fu+Prop combination group experienced a more significant inhibitory effect on HIF-1α and NF-κB (P<0.05). The four chemotherapy schemes yielded a significant inhibitory effect on Ki67 (P<0.05), and combination therapy generated a more pronounced inhibitory effect than monotherapy. Compared with the Ctrl group, the intestinal mucosa in the 5-Fu group and 5-Fu+Ox group exhibited a disordered arrangement and destroyed architecture. Intestinal mucosa damage in the Prop group and the 5-F+Prop combination group was reduced (Figure 4).

## Discussion

The occurrence and development of CRC result from the complex interplay between sequential gene mutation and epigenetic change [14,15]. Mutations in genes and their respective signal transduction pathways lead to changes in the cell cycle and ultimately affect different cell behaviors. Indeed, timely diagnosis and effective precise treatment are the key to improving the success rate of tumor treatment and the prognosis of patients [16]. After radical resection of CRC, systematic treatment, including chemotherapy, is an effective method to improve the survival rate. The heterogeneity of tumors and the complex microenvironment account for the different responses to chemotherapy schemes. Accordingly, some insensitive patients may progress rapidly after surgery after treatment with traditional adjuvant chemotherapy. In this study, we analyzed the main factors affecting tumorigenesis in the CRC-PDX model, the time to tumorigenesis, and the feasibility of adjuvant therapy after surgery, compared the biological characteristics between tumorigenesis and PDX model, conducted drug sensitivity experiments using the PDX model.

For the clinical application of the CRC-PDX model, the success rate of tumor tissue transplantation in mice is essential. Transplantation failure is another limiting factor for the clinical application of the tumor PDX model. The tumorigenicity rate of PDX transplanted tumors was 25% to 60% [5]. In this study, the fresh tissues obtained from patient surgery were inoculated into immunodeficient NSG mice: one of the most suitable models for human tissue transplantation, to establish a CRC-PDX model, yielding a tumorigenicity rate of 62%. In previous studies, the average tumorigenesis rate of CRC-PDX was about 70% [17]. The tumorigenicity rate is determined by the animal host used and the source of tumor tissue [5]. This study showed that the tumorigenesis rate was related to the degree of malignancy of the primary tumor, such as tumor stage and cell differentiation.

Indeed, in the PDX tumor model, the patient-derived primary tumor tissue is grown in the tissue environment of the mouse [18,19]. It has been established that the PDX tumor transplantation model can reproduce tumor heterogeneity of patients at a high level and has good predictability of clinical chemotherapy effect [5,20]. The biological stability of the PDX tumor model is an important feature; it has been reported that the CRC-PDX model exhibits good performance in replicating the biological characteristics of the original tumor [21-23] in terms of histopathology, oncogene expression, and sensitivity to chemotherapy drugs, emphasizing that it is an ideal and reliable biological transformation model. PDX technology makes up for the shortcomings of cell line-derived xenograft (CDX) and it is important for early clinical evaluation, treatment, and prognosis of tumors. It is expected to bring significant breakthroughs for the individualized treatment of tumor patients. This study used NSG mice to establish a CRC-PDX model and grow to the F3 generation. The histological structure and immunohistochemical expression of HIF-1α, NF-κB, and ki67 shown in HE staining were consistent with the patient’s primary tumors. It is suggested that in the PDX model and passage process, the histomorphology and molecular phenotype of tumors in P and F3 generation remain stable in genetics and exhibit good performance in retaining characteristics of primary tumors.

Interestingly, the PDX model achieved completely different results than the traditional CDX model in predicting the efficacy of anti-tumor drugs. Drugs with good efficacy on the CDX model in the past often exhibited decreased efficacy after being applied to the human body, and the conversion success rate from preclinical to clinical trials was less than 10% [19]. Microfluidic models reflect the situation of human tumors and promote the development of chip-on-chip, a type of in vitro platform that can accurately simulate various characteristics of the cancer microenvironment, such as microvascular structure, but its consistency with cancer patients has not been rigorously tested [24]. In contrast, using PEX models based on samples from cancer patients is still the most reasonable and feasible choice in reflecting the true structure of cancer. Patient-derived organoids are cultured from cancer cells in the patient and are a cost-effective tool for preserving cancer information. However, in vitro models are not ideal for testing new therapies because they cannot reflect many in vivo characteristics, such as pharmacokinetic performance, in preclinical drug testing [25]. In contrast, the PDX model can better reflect the characteristics of cancer and stimulate the progression and evolution of tumors in human patients. The PDX model has produced the most convincing preclinical results and is considered one of the most promising models to address the challenges that clinical physicians face, including identifying prognostic biomarkers, exploring the impact of tumor heterogeneity on tumor progression, and evaluating new drugs [26].

Importantly, the PDX-CRC model can be used as a "surrogate" model for individualized anticancer drug screening. The predicted sensitivity and specificity rates of CRC chemotherapy verified by the PDX model were higher than 90% [27]. In one prospective study, the surrogate mouse PDX model of 14 patients was used to study and screen the effective treatment plans among 232 treatment plans containing 63 drugs, and then 17 of the most effective treatment plans for PDX models were used to treat 11 patients, of which 15 treatment plans yielded lasting partial remission effect [28]. A recent multicenter study showed that PDX transplanted tumors of advanced gastrointestinal tumors retain the nature of primary tumors, and PDX model prediction is consistent with clinical drug response, which is suitable for personalized drug treatment of advanced gastrointestinal tumors [29]. In this study, four chemotherapy regimens yielded anticancer effects on a rat model of advanced malignant CRC-PDX, and their combination was more effective than the monotherapy. Propofol is an anesthetic that has an anticancer effect and can enhance the effect of other anticancer drugs. In addition, propofol can relieve diarrhea caused by chemotherapy and protect intestinal mucosa. Interestingly, it has been shown that Propofol has anti-inflammatory characteristics and immunomodulatory effects, which may have beneficial effects on inhibiting cancer recurrence [30,31]. Propofol may inhibit tumor cells by binding to the GABA receptor on the surface of tumor cells to increase the intensity of chloride current [32]. Propofol can downregulate HIF-1α in tumor cells and inhibit tumor angiogenesis [33] and tumor growth in mice [34,35]. Intriguingly, we found that propofol alone could downregulate the expression of HIF-1α in tumor tissue of the CRC-PDX mouse model, which was enhanced after combination with 5-Fu, although the specific mechanism is unclear, warranting further study.

Given that the number of patients in this study was small and the variation was significant during multivariate analysis, only univariate analysis was used. The homology between tumor tissue and primary tumor tissue of the PDX model was assessed only by tissue morphology and CRC tumor-related protein expression. A research team previously found that the HIF-1α activator yielded a protective effect against sepsis-induced intestinal mucosal damage [36]. In this study, propofol downregulated HIF-1α in tumor tissue. However, whether HIF-1α mediates the protective effect of propofol on intestinal mucosa remains obscure.

## Conclusions

To sum up, as the closest tumor animal model to clinical research, the CRC-PDX model is highly consistent with the primary tumor in terms of pathological characteristics and biological characteristics, which is of great significance for the evaluation of chemotherapeutic drug sensitivity. The malignant degree of the CRC primary tumor and the preoperative CEA level of patients are influencing factors of PDX tumorigenesis rate. The anesthetic propofol can inhibit the growth of CRC and reduce intestinal mucosal damage caused by chemotherapy and diarrhea. Further studies are required to explore the underlying mechanisms.
